# DNALI1 deficiency causes male infertility with severe asthenozoospermia in humans and mice by disrupting the assembly of the flagellar inner dynein arms and fibrous sheath

**DOI:** 10.1038/s41419-023-05653-y

**Published:** 2023-02-15

**Authors:** Huan Wu, Yiyuan Liu, Yuqian Li, Kuokuo Li, Chuan Xu, Yang Gao, Mingrong Lv, Rui Guo, Yuping Xu, Ping Zhou, Zhaolian Wei, Rong Hua, Xiaojin He, Yunxia Cao

**Affiliations:** 1 Reproductive Medicine Center, Department of Obstetrics and Gynecology, the First Affiliated Hospital of Anhui Medical University, No. 218 Jixi Road, 230022 Hefei, Anhui China; 2 NHC Key Laboratory of Study on Abnormal Gametes and Reproductive Tract, Anhui Medical University, No. 81 Meishan Road, 230032 Hefei, Anhui China; 3 Key Laboratory of Population Health Across Life Cycle, Anhui Medical University, Ministry of Education of the People’s Republic of China, No. 81 Meishan Road, 230032 Hefei, Anhui China; 4 Anhui Province Key Laboratory of Reproductive Health and Genetics, Anhui Medical University, No. 81 Meishan Road, 230032 Hefei, Anhui China; 5 Anhui Provincial Engineering Research Center of Biopreservation and Artificial Organs, Anhui Medical University, No. 81 Meishan Road, 230032 Hefei, Anhui China; 6 Anhui Provincial Institute of Translational Medicine, Anhui Medical University, No. 81 Meishan Road, 230032 Hefei, Anhui China

**Keywords:** Infertility, Disease genetics

## Abstract

The axonemal dynein arms (outer (ODA) and inner dynein arms (IDAs)) are multiprotein structures organized by light, intermediate, light intermediate (LIC), and heavy chain proteins. They hydrolyze ATP to promote ciliary and flagellar movement. Till now, a variety of dynein protein deficiencies have been linked with asthenospermia (ASZ), highlighting the significance of these structures in human sperm motility. Herein, we detected bi-allelic *DNALI1* mutations [c.663_666del (p.Glu221fs)], in an ASZ patient, which resulted in the complete loss of the DNALI1 in the patient’s sperm. We identified loss of sperm DNAH1 and DNAH7 rather than DNAH10 in both *DNALI1*^*663_666del*^ patient and *Dnali1*^*−/−*^ mice, demonstrating that mammalian DNALI1 is a LIC protein of a partial IDA subspecies. More importantly, we revealed that DNALI1 loss contributed to asymmetries in the most fibrous sheath (FS) of the sperm flagellum in both species. Immunoprecipitation revealed that DNALI1 might interact with the cytoplasmic dynein complex proteins in the testes. Furthermore, DNALI1 loss severely disrupted the transport and assembly of the FS proteins, especially AKAP3 and AKAP4, during flagellogenesis. Hence, DNALI1 may possess a non-classical molecular function, whereby it regulates the cytoplasmic dynein complex that assembles the flagella. We conclude that a DNALI deficiency-induced IDAs injury and an asymmetric FS-driven tail rigid structure alteration may simultaneously cause flagellum immotility. Finally, intracytoplasmic sperm injection (ICSI) can effectively resolve patient infertility. Collectively, we demonstrate that *DNALI1* is a newly causative gene for AZS in both humans and mice, which possesses multiple crucial roles in modulating flagellar assembly and motility.

## Introduction

Sperm motility is critical for male fertility since the ejaculated sperm must self-propel its travel along the long female reproductive system to fertilize the egg and initiate life. Asthenozoospermia (ASZ) is a frequent form of primary male infertility, whereby over 40% of cases present some defects in sperm motility [1–3]. Sperm flagellum is uniquely designed for its key biological role of beating wave formation. This highly conserved and microtubular organelle is precisely organized by a core cytoskeletal structure known as the axoneme, as well as a variety of peri-axonemal elements [4]. The axoneme is permanently located in the center of the flagellum and consists of nine peripheral doublet microtubules (DMTs), which circumferentially surround a central microtubular pair (CP) to form the classical ‘9 + 2’ arrangements. Mechanical links formed by radial spokes, dynein arms, and nexin-dynein regulatory complex (N-DRC) maintain the integrity and coordinate the dynamics of these microtubules [5]. In addition, several accessory subunits, comprising the nine outer dense fibers (ODFs), mitochondrial sheath (MS), and fibrous sheath (FS), layer by layer encompass different segments of the central axoneme [6]. Based on these discrete subcellular structures along the longitudinal axis, the flagellum can be classified into three segments, namely, the middle, principal, and end pieces. ODFs are surrounded by the spiral MS in the middle region and by FS in the principal region. Distal to MS, FS is a tapered cylinder that consists of two longitudinal columns (LCs) with nearly symmetrical distribution, and several connecting circumferential ribs (CRs) distributed between the LCs [7].

The discovery of axonemal dyneins provides some insight into the mysterious mechanism of flagellar bending motion [8]. Axonemal dyneins are highly complicated molecular motors that contain a variable number of heavy (DHC), intermediate, light, and light intermediate chain (LIC) polypeptides that vary based on their molecular weight and cellular activity [9]. Given their distinct position within the axoneme, dynein motors can be further subdivided into two classes: outer dynein arms (ODAs) and inner dynein arms (IDAs). Defects within any axonemal dynein arms seriously impair ciliary motility in *Chlamydomonas* [10, 11]. In mammals, biallelic mutations in multiple genes involved in DHC biogenesis, such as *DNAH1* (MIM: 603332) [12], *DNAH2* (MIM: 603333) [13], *DANH7* (MIM: 610061) [14], *DANH8* (MIM: 610061) [15], *DANH10* (MIM: 605884) [16], and *DANH17* (MIM: 603340) [17], are reported to cause male sterility in humans and mice, which is characterized by severely diminished sperm movement and multiple morphological abnormalities of the flagella (MMAF). These findings not only emphasize the crucial role of DHC in flagellar assembly but also raises the question of how other chains function in unison during spermatogenesis.

*Dynein axonemal light intermediate chain 1* [*DNALI1* (MIM: 602135)] encodes a LIC subunit of IDA in mammals [18, 19]. The orthologue *DNALI1* gene, known as p28, was originally identified in *Chlamydomonas* and is known to interact with DHC2 during axonemal IDA assembly [20]. Notably, its orthologue genes in sea urchins and mice were subsequently identified as having a potential role in modulating sperm motility [19, 21]. This evidence suggested an interesting possibility that DNALI1 is indispensable in sperm flagellogenesis; however, the specific mechanism remains inconclusive. Herein, we detected a homozygous frameshift mutation in *DNALI1* in an ASZ patient from a Chinese consanguineous family and explored the effect of this loss-of-function (LOF) mutation in flagellar integrity and motility. Most importantly, similar to the LOF *DNALI1* mutation in our ASZ patient, the *Dnali1*-knockout (KO) male mice also displayed immotile sperm, featured by subcellular defects within the flagellar IDA and FS. Furthermore, we analyzed the potential roles of DNALI1 during spermatogenesis using the KO mice model and attempted to identify the intracytoplasmic sperm injection (ICSI) outcomes of the *DNALI1*-mutant patient. Our interactome data provides insights into the biological function of DNALI1 in mammalian spermatogenesis and highlights that *DNALI1* is a novel causative gene for AZS in both humans and mice.

## Materials and methods

### Study patients

Overall, 65 idiopathic ASZ patients, including two pairs of brothers, from 63 consanguineous Chinese families, were recruited from the First Affiliated Hospital of Anhui Medical University. Participants with abnormalities in any of the following items including somatic chromosome karyotype, genomic azoospermia factor deletions, serum sex hormone levels, and scrotal ultrasonography were excluded from the analysis. This investigation received ethical approval (approval nos. PJ2017-11-10 and PJ2020-13-10) from the aforementioned institution and, received documented informed consent from all subjects prior to the initiation of the study. All the studies were carried out in accordance with the Declaration of Helsinki and approved by the institutional ethics review board.

### Genetic analysis

Whole-exome sequencing and bioinformatic analysis were performed as reported in a prior publication [22]. Briefly, genomic DNA extraction and whole exome enrichment were performed sequentially, following standardized protocols. Subsequently, high-throughput sequencing of the captured DNA was processed on the HiSeq X-TEN or NovaSeq 6000 platform (Illumina, San Diego, CA). The standard assembly (Burrows–Wheeler Aligner, http://bio-bwa.sourceforge.net/), calling (Genome analysis Toolkit, https://gatk.broadinstitute.org/hc/en-us), and annotation (ANNOVAR, https://annovar.openbioinformatics.org/en/latest/) employed follow-up bioinformatic analysis. Lastly, sanger sequencing was performed to validate candidate mutations and corresponding origins.

### Sperm characteristics study

We collected fresh semen samples from ASZ and control males during routine laboratory examinations following the World Health Organization (WHO) guidelines (5th Edition) [23]. The samples were obtained via masturbation following 3–7 days of sexual abstinence and were liquified for 30 min at 37 °C prior to evaluation. We assessed semen volume, sperm content, and motility, and this process was replicated for all routine examinations.

### Western blot (WB) analysis

WB was carried out as described previously [24], with slight modification. In brief, total protein extraction was done in a lysis buffer (7 M urea, 2 M thiourea, 2% (w/v) DTT) with 1% (v/w) protease inhibitor mixture (Pierce Biotechnology). Equal protein amounts were then separated in an SDS–PAGE, prior to transfer to PVDF membranes, which next underwent blocking in 5% nonfat milk in TBST for 2 h at room temperature (RT), prior to overnight (ON, (>12 h) exposure to primary antibodies at 4 °C. Following three TBST rinses, the membranes were treated with secondary antibodies for 2 h, and subsequently, with the SuperSignalWest Femto Chemiluminescent Substrate WB detection system (Thermo Scientific) to visualize protein bands.

### Immunofluorescence (IF) staining of paraffin-embedded sections, testicular single-cell smear, and spermatozoa

Paraffin-embedded sections underwent deparaffinization and rehydration, followed by three 10 min PBS rinses. Antigen was then extracted by boiling the slides for 10 min in 10 mM citrate buffer (pH 6.0) in a microwave. Following three 10 min PBST (0.1% Tween-20 in PBS) rinses, the slides were ready for IF staining.

Immunostaining of testicular single-cell smears was carried out based on a previously published protocol [25]. In short, testes digestion was carried out in collagenase IV for 10 min at 37 °C, followed by trypsin-EDTA solution for 15 min at 37 °C, prior to a brief rinse in PBS. Following a few PBS rinses, the cell pellets were dissolved in PBS, prior to fixation in the same quantity of fixation buffer (4% PFA). Lastly, the slides were left on a bench to air-dry.

To test sperm samples, we spread sperm directly on a slide and allowed them to air-dry, subsequently, the samples were fixed in 4% PFA buffer, and stained with IF antibodies.

To conduct IF, all samples were thrice rinsed in PBST, then 1 h-treated with 1% BSA diluted in PBST, prior to an ON exposure to targeted antibodies at 4 °C. This was followed by a 2 h treatment with secondary antibody at RT, with subsequent Hoechst33342 staining for 5 min. Lastly, the slides were PBS-rinsed and mounted with glycerol, prior to visualization with an LSM800 confocal microscope (Carl Zeiss AG), equipped with ZEN2013 software (Carl Zeiss AG).

### Antibodies

DNALI1 (Abnova, H00007802-B01P, mouse), β-TUBULIN (Affinity, AF7011, Rabbit), AKAP4 (FineTest, FNab00256, Rabbit), GAPDHS (Sigma-Aldrich, HPA042666, Rabbit), DNAH1 (Thermo Fisher, PA5-57826, Rabbit), DNAH12 (Thermo Fisher, PA5-63952, Rabbit), DNAH7 (Bioss, bs-11023R, Rabbit), DNAH10 (Bioss, Bs-11022R, Rabbit). Anti-DRC1 and anti-AKAP3 were gifts from the Liu laboratory [26] and the Qi Laboratory [27], respectively.

### Transmission electron microscopy (TEM)

TEM analysis was done as reported earlier [28]. The samples underwent fixation in 1% osmium tetroxide, followed by dehydration in a series of ethanol (50%, 70%, 90%, and 100%) and 100% acetone solutions. Once they underwent infiltration with acetone and SPI-Chem resin and embedding via Epon812, the samples were cut using ultra-microtome and treated with uranyl acetate and lead citrate staining. Visualization and image capture was done with a cryoelectron microscope Talos L120C G2 (Thermo Fisher, USA).

### Hematoxylin and eosin (H&E) staining

Human sperm was spread on glass slides, followed by a 10 min fixation in 4% PFA. Following a PBS rinse, the slides underwent H&E staining, following standard protocols.

### Immunoprecipitation (IP)

To conduct IP, we accumulated 8 ml of human sperm, and rinsed thrice with PBS, prior to lysis in RIPA buffer (P0013B, Beyotime) with phosphatase inhibitor cocktail A,50X (P1082, Beyotime) and phenylmethanesulfonyl fluoride (ST505, Beyotime). Both human sperm and mice testes (200 mg) underwent homogenization in 2 ml lysis buffer on ice for 40 min, prior to centrifugation at 12,000 × *g* for 20 min at 4 °C. The supernatant was saved in a new tube for IP analysis as follows: First, it underwent ON incubation with anti-DNALI1 at 4 °C, followed by treatment with protein A/G (Bimake, B23202) beads for 2 h at 4 °C. Subsequently, the precipitants were twice rinsed in IP buffer (20 mM Tris, pH 7.4, 2 mM EGTA and 1% NP-40), and the immune complexes were eluted with sample buffer and 1% SDS for 10 min at 95 °C, prior to analysis via immunoblotting or mass spectrometry (MS).

### MS

The IP precipitants were separated using the PAGE-gel and stained via AgNO_3_. The gels were cut following trypsin digestion. The EASY-nanoLC 1200 system (Thermo Scientific), equipped with an Orbitrap Q Exactive HFX mass spectrometer (Thermo Scientific), via a nanospray ion source, was employed for LC-MS/MS analysis. Tryptic peptide mixtures were dissolved in 0.1% formic acid (FA) in LC-grade water in an analytical column (75 μm × 25 cm, C18 column, 1.9 μm, Dr. Maisch). A 95-min linear gradient (3–5% B for 5 s, 5–15% B for 40 min, 15–28% B for 34 min and 50 s, 28–38% B for 12 min, 30–100% B for 5 s, and 100% B for 8 min) was applied during the buffer (0.1% FA (buffer A) and 80% ACN, 0.1% FA (buffer B)) incubation using a high-resolution MS pre-scan, and a mass range of 350–1500. The normalized collision energy for elevated energy collision-driven dissociation (HCD) was adjusted to 28, and the resulting fragments were identified using a resolution of 15,000. All ions chosen for fragmentation were eliminated for 30 s via dynamic exclusion. Data processing was done via the MaxQuant software (version 1.6.14), and the mouse reference proteome was retrieved from the UniProt database (release 2021.04) using standard variables.

### ICSI procedures

ICSI was performed as previously described [22]. In brief, oocytes were retrieved following controlled ovarian stimulation. In the meantime, viable sperms were selected via evaluation of the sperm tail elasticity, prior to injection into the MII oocytes using ICSI pipettes. Next, we transferred the day 5 embryo to the partner and confirmed successful pregnancy via ultrasonographic examination on the next 7th week post-implantation.

### Animals

Our animal care and protocols strictly followed the criteria set by the Institutional Animal Care and Use Committee of Cyagen Biosciences Inc. Moreover, all animal protocols received ethical approval from the institution (Approval No. KOAIB210329DY1).

The mice were housed in a pathogen-free environment at 20–22 °C, under a 12 h light/dark cycle, 50–70% humidity, and free access to food and water. The mice were treated humanely, and suffering of any kind was kept to a minimum. When needed, mice sacrifice was done via cervical dislocation. Male mice at the age of 8–10 weeks were subjected to obtain testes, sperm, and brain tissue for phenotypic analysis. No statistical methods were used to predetermine the sample size. Mice were randomly allocated to experimental groups. No blinding method was used for animal studies. There were no animal exclusion criteria. All animals were killed for tissue analyses by the end of the study.

### Generation of the *Dnali1* knockout mouse model and genotyping

We established the *Dnali1*-knockout mice using CRISPR/Cas9. In short, *Dnali1* exon 3-5-targeting single-guide RNAs (sgRNAs) were synthesized using the following primers: 5’-TGCATAAGACTATTAGGTGG*AGG*-3’ and 5’-GTCATGGGAGTCCTCTCGGC*AGG*-3’. The Cas9 and sgRNA plasmids underwent linearization with AgeI and DraI, respectively, prior to purification using a MinElute PCR Purification Kit (Qiagen, Duesseldorf, Germany). The Cas9 mRNA was established using A MESSAGE mMACHINE T7 Ultra Kit (Ambion, TX, USA), and the purified sgRNA was generated using the MEGA Shortscript and Clear Kit (Ambion). Wild-type (WT) C57BL/6 superovulated females were then mated with C57BL/6 males to produce zygotes for Cas9 mRNA and sgRNA administration.

Subsequently, edited founders carrying the *Dnali1*-edited gene were then mated with WT mice for at least three generations to minimize the potential effects of off-target gene editing. The resultant offspring genotypes were validated via PCR using the following primers: F, 5’-CATGGGGTCTCAGTATGGTTGTAT-3’; R1, 5’- AGTGAATTCTGTGCTGGAGGAATA-3’; R2, 5’-TAATCCCTTCTCTTTCCCATCTGGT-3’.

### Mice sperm analysis

Mice sperm analysis was performed using computer-assisted sperm analysis (CASA) as previously reported [29]. In short, the distal cauda region of the right epididymis was clamped and excised, followed by rinsing with warm PBS, and placement in an Eppendorf tube with fresh human tubal fluid (HTF) media (Millipore) and 10% FBS at 37 °C. Subsequently, clamping was reversed, and the cauda was punctured using a scalpel to facilitate sperm to transfer to the medium for 5 min at 37 °C. The sperm was then diluted with more medium, and 10 μl sperm suspension was used to assess sperm motility using a computer-assisted program (Hamilton Thorne Research Inc.).

### Histological analysis

Mice testes or epididymal tissues were harvested from at least three mice from each category, prior to a 24 h fixation in modified Davidson’s fluid, and storage in 70% ethanol. A series of ethanol was employed to dehydrate the samples, prior to paraffin-embedding, and slicing to generate 5 μm-thick sections, mounted on glass slides. The slides were then deparaffinized, and stained with H&E, prior to histological analyses.

### Statistical analysis

We conducted all experimentations three times or more. Intergroup differences were compared via one-way ANOVA or unpaired two-tailed *t*-tests, and are presented as mean ± s.d. The variance was similar between the groups that were being statistically compared. GraphPad Prism 9.0 was employed for all data analyses. Not significant (NS); *P* > 0.05, *P < 0.05, **P < 0.01, ***P < 0.001, and ****P < 0.0001 were all significant.

## Results

### Homozygous loss-of-function mutation in *DNALI1* identified as a potential pathogeny for human AZS

**Fig. 1:**
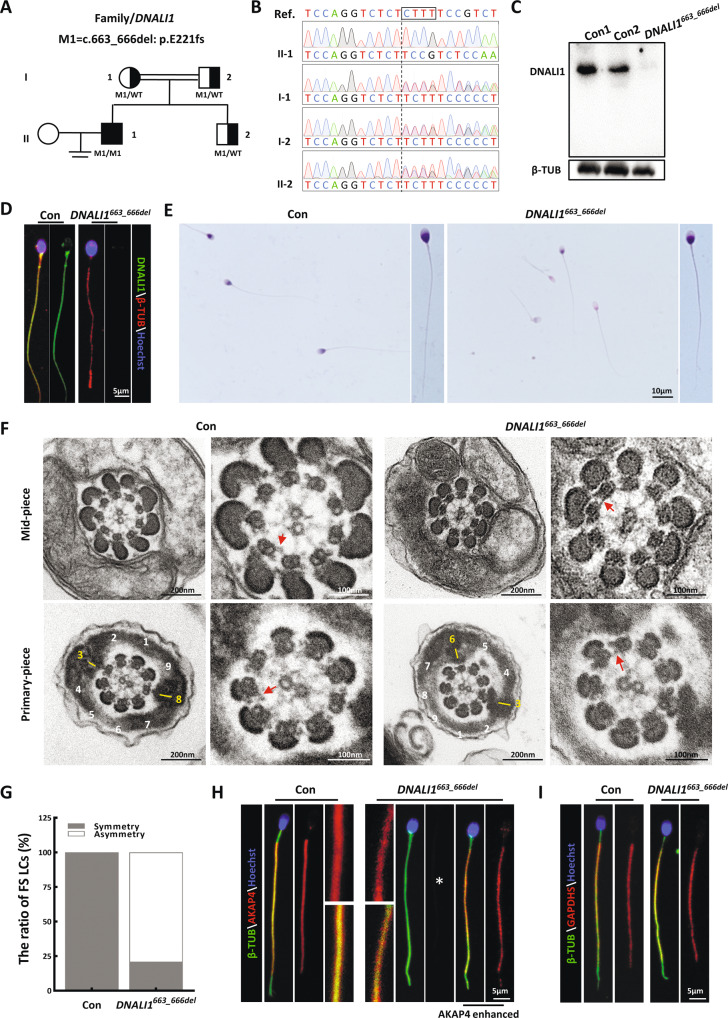
The DNALI1 loss leads to the asymmetrical development of sperm fibrous sheaths in humans. **A** Pedigree analysis of the family affected by bi-allelic *DNALI1* mutations, as identified by WES. The black-filled squares represent the infertile males of this family. **B** The WES-identified mutations are further confirmed via Sanger sequencing. The black box marks the missing sequence. **C** DNALI1 is missing in spermatozoa from the patient. **D** Spermatozoa from a fertile control male and the *DNALI1*^*663_666del*^ patient are stained with anti-DNALI1 and anti-βTUBULIN (β-TUB). **E** Light microscopy revealed spermatozoa with normal morphology from the *DNALI1*^*663_666del*^ patient and a control male. **F** TEM images of the flagellar cross-sectional structure of a control human and patient. The red arrow represents an obvious IDA. The numbers highlight the quantity of peripheral microtubules. A yellow dash represents the LCs, which are diagonally positioned in the control spermatozoa. **G** TEM cross-sections of the principal piece of sperm are quantified and categorized according to the LC symmetry. **H** and **I** Immunofluorescence (IF) staining against AKAP4 and GAPDHS. The asterisk indicates a significant decrease in AKAP4 protein expression.

Several causative genes, including the *DNAH*, *CFAP*, and *DRC* family members [14, 26, 28, 30–35], were identified in our cohort. We reanalyzed the remaining exome sequencing data, and detected a homozygous frameshift mutation in *DNALI1* [c.663_666del (p.Glu221fs)] in an additional subject (AC050) (Fig. 1A). Spermatozoa of the otherwise healthy proband were entirely immotile, but exhibited normal flagellar morphology (Table 1). Sanger sequencing confirmed that these biallelic mutations originated from heterozygous asymptomatic parents, suggesting an autosomal recessive mode of inheritance (Fig. 1B). This mutation, with a deletion of four bases, was estimated to promote a translational frameshift and a premature stop codon during DNALI1 translation (Fig. S1A). Immunoblotting and immunostaining analyses were conducted to assess the pathogenicity of this putative LOF mutation. The semen samples of AC050 were obviously devoid of DNALI1 without a truncated form (Fig. 1C). Furthermore, DNALI1-immunostaining foci were absent from the mutant sperm flagella (Fig. 1D). These results demonstrated that this LOF mutation promoted a complete loss of DNALI1 in the affected sperm samples.

**Table 1:** Clinical characteristics and ICSI outcomes of the DNALI1-mutant patient.

	Patient
	AC050
*Gene*	*DNALI1*
cDNA mutation^a^	c.663_666del
Amino acid alteration^b^	Glu221fs
Exon	5
Zygosity	Homozygous
Mutation type	Frameshift deletion
Allele frequency	
1KGP	NA
ExAC	NA
gnomAD	NA
*Semen parameters*	
Semen volume (ml)	3.2
Sperm concentration (10^6^/ml)	23.5
Progress motility (%)	0
Motility (%)	0
Normal morphological spermatozoa (%)	47
*ICSI treatment*	
No. of ICSI cycles	1
No. of oocytes injected	8
Fertilization rate (%)	8
Cleavage rate (%)	8/8 (100)
8 cells embryo development rate (%)	4/8 (50)
Blastocyst development rate (%)	4/8 (50)
Number of embryos transferred	1
Implantation rate (%)	1/1 (100)
Clinical pregnancy	Y
Live birth	Boy

*1KGP* 1000 Genomes Project, *ExAC* Exome Aggregation Consortium, *gnomAD* Genome Aggregation Database, *NA* not available, *Y* yes.

^a^*DNALI1* GenBank accession No. NM_003462.5.

^b^Full-length DNALI1 protein has 258 amino acids.

### DNALI1 deficiency disrupts the assembly of the flagellar fibrous sheath in humans

H&E staining revealed that most mutant spermatozoa appeared normal (Fig. 1E). To characterize the *DNALI1*^*663_666del*^-mediated regulation of subcellular sperm flagellar morphology, we assessed the cross-sectional flagellar ultrastructures. Relative to the symmetrical FS in the control sperm, asymmetric FS was frequently observed in the mutant flagella. Being a prominent component of FS, which surrounds the ODFs, the LC of the control sperm replaced ODF #3 and #8 in the principal piece. However, over 79% of the mutant FS presented irregular LC localization (Fig. 1F and G), suggesting a possibility that the DNALI1 deficiency disrupted the proper assembly of FS during spermatogenesis. This was further confirmed by our IF data, which revealed that the AKAP4-immunostained foci were relatively absent in the flagella lacking DNALI1 (Fig. 1H). In contrast, the unaltered signal of GAPDHS, a spermatogenic glycolytic enzyme located within FS, was evident in the mutant samples (Fig. 1I).

### DNALI1 is a fundamental component of IDAs that determines the flagellar localization of corresponding DHC subunits

**Fig. 2:**
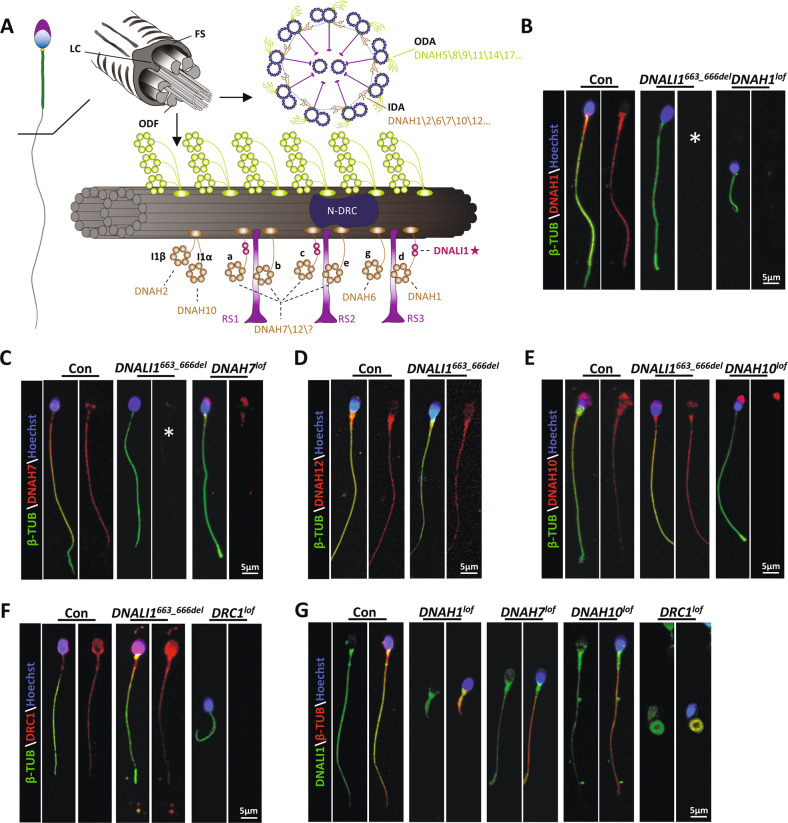
Location of IDA components in the human flagellum. **A** A schematic diagram of the positions of the dynein proteins docks on the A-tubule of the microtubule doublet (green) in the 96 nm axonemal repeat, according to the prior structural information on *Chlamydomonas* and Sea urchin sperm flagella. **B**–**E** IF illustrating various dyneins components’ locations on the human sperm flagella. A loss of DNALI1 leads to the mislocation of DNAH1(**B**) and DNAH7(**C**). Asterisks indicate the miss of DNAH1 and DNAH7, respectively. **F** IF staining against DRC1 (a component of the N-DRC structure adjacent to IDA). **G** The spermatozoa from male infertile patients with indicated protein deficiency are stained with anti-DNALI1.

In the *Chlamydomonas* flagellum, the DNALI1 homologous protein is speculated to be localized within the subspecies a, c, and d of IDAs [19, 36] (Fig. 2A). Since no obvious ultrastructural IDA abnormalities were detected in the cross-sectioned mutant flagellar specimen (Fig. 1F), we speculated that DNALI1 deficiency may only lead to the disassembly of some DHC subunits rather than impair the entire IDAs. To further identify the DHC subunits that interact with DNALI1, we performed IP with sperm protein extracts from the ejaculated semen of fertile men. MS identified both DNAH1 and DNAH12 as potential interacting proteins of DNALI1 (Table S1). Next, IF experiments were conducted using sperm from the proband. Based on our data, DNAH1 was completely absent in the *DNALI1*^*663_666del*^ sperm, while no significant DNAH12 deficiency was observed (Fig. 2B and D), suggesting a hypothesis that DNAH12 might interact with multiple proteins including DNALI1 during flagellogenesis. In addition, DNAH7, a component related to the flagellar subspecies c in *Chlamydomonas* [37], was completely lost in the *DNALI1*^*663_666del*^ sperms (Fig. 2C). Being a component of subspecies I1α, DNAH10 was not affected by the loss of DNALI1 (Fig. 2E). We also detected DNALI1 in the DNAH1\7\10-deficient sperm collected from our previous studies [14, 16, 31]. The DNALI1 signal was persistently visible in the absence of any DNAH1, DNAH7, or DNAH10 (Fig. 2G). In addition, DRC1, a cornerstone subunit of N-DRC, was evident in the *DNALI1*^*663_666del*^ flagella (Fig. 2F). Similarly, the DNALI1 signal remained within the flagella lacking DRC1 [26] (Fig. 2G). These findings collectively indicated that DNALI1 was indispensable for the flagellar assembly of DNAH1, DNAH7, and, potentially, DNAH12. Conversely, DNAH1, DNAH7, and DNAH10 were not required for the flagellar distribution of DNALI1. In addition, although there was a strong correlation between IDAs and DRC, no structural interdependence was evident between DNALI1 and DRC1.

### Deletion of *Dnali1* in mice impairs the subcellular structure of sperm flagella, causing severe ASZ

**Fig. 3:**
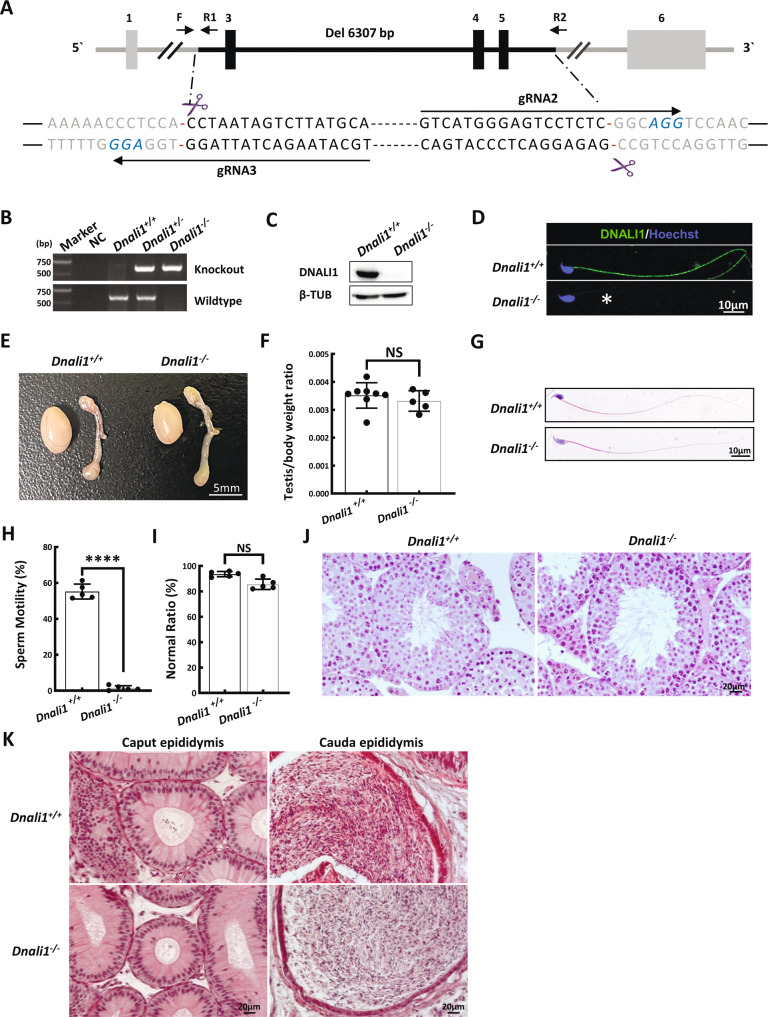
*Dnali1*^*−/−*^ males display asthenozoospermia (ASZ). **A** A summary of the CRISPR/Cas9 knockout targeting approach. **B** The PCR identification of mouse genotypes. Primer sites are shown in (**A**). F\R1 is used for wildtype sequence and F\R2 for knockout sequence. C Western blotting verifying that testis samples from *Dnali1*^*−/−*^ mice do not produce a band of the expected size (28 kDa). β-TUBULIN (β-TUB) was used as a normalization control. D IF analysis of spermatozoa from the *Dnali1*^*+/+*^ and *Dnali1*^*−/−*^ mice using an anti-DNALI1 antibody. Asterisks indicate the complete loss of DNALI1. **E** Testes from 9-week-old *Dnali1*^*+/+*^ and *Dnali1*^*−/−*^ mice. **F** Mean testis weight adjusted with body weight, Data are presented as means ± SEM (*n* = 8 vs. 5; NS, *P* > 0.05, Student’s *t*-test). **G** H&E-stained spermatozoa from the *Dnali1*^*+/+*^ and *Dnali1*^*−/−*^ mice. **H** Mean frequencies of the motile sperm in the *Dnali1*^*+/+*^ and *Dnali1*^*−/−*^ mice, (*n* = 5 vs. 5; ****P < 0.0001, Student’s *t*-test). **I** Frequencies of sperm with aberrant morphological profiles in the *Dnali1*^*+/+*^ and *Dnali1*^*−/−*^ mice (*n* = 5 vs. 5; NS, *P* > 0.05, Student’s *t*-test). **J** and **K** H&E-stained testis (**J**) and epididymis (**K**) sections from the *Dnali1*^*+/+*^ and *Dnali1*^*−/−*^ mice.

DNALI1 is an evolutionarily conserved protein between humans and mice. In fact, approximately 96% amino acids of DNALI1 are identical in both species (Fig. S1B). We established the *Dnali1*-KO mice using the CRISPR-Cas9 genome editing technique to confirm that *DNALI1*-deficiency is indeed a novel causative mutation for human AZS. A stable mutant mouse line carrying a three exons (exons 3–5) deletion in *Dnali1* was obtained (Fig. 3A). The mouse DNA fragment deletion was then confirmed via PCR (Fig. 3B). WB and IF were further used to validate the complete loss of DNALI1 protein in the testes and sperm of *Dnali1*^−/−^ mice (Fig. 3C and D).

To explore whether *Dnali1*^−/−^ mice have similar reproductive phenotypes as our AZS patient, we examined spermatogenesis in *Dnali1*^−/−^ males. The appearance and weight of the testis and epididymis were comparable between *Dnali1*^−/−^ and *Dnali1*^+/+^ male mice (Fig. 3E and F). CASA analyses revealed that all epididymal sperms were immotile in the *Dnali1*^−/−^ mice (Fig. 3H, Movies 1, 2). Moreover, spermatozoa morphology appeared completely normal in the *Dnali-*KO mice (Fig. 3G and I). To determine whether DNALI1 deficiency induced spermatogenic defects, we explored the testicular and epididymis sections. No difference was observed between the two genotypes (Fig. 3J and K).

**Fig. 4:**
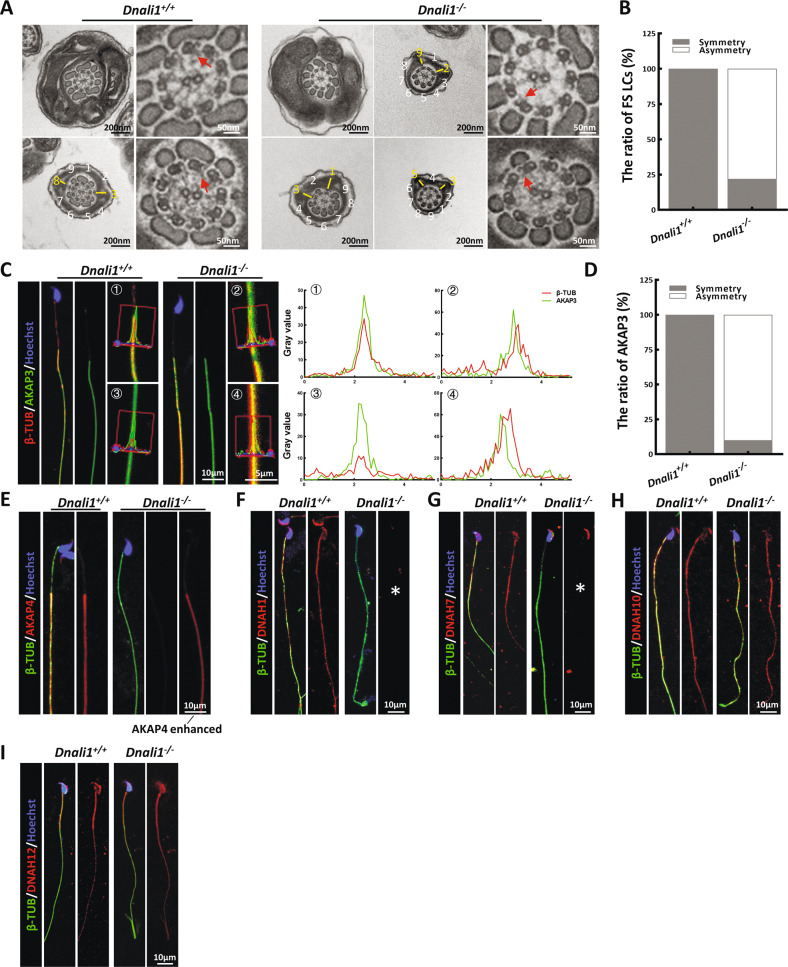
The LCs position of the *Dnali1*^*−/−*^ spermatozoa is asymmetrical and the IDA components are incomplete. **A** and **B** The flagellar cross-sectional structure of *Dnali1*^*+/+*^ and *Dnali1*^*−/−*^, as evidenced by TEM. The red arrow indicates an obvious IDA. The numbers represent the quantity of peripheral microtubules. A yellow dash marks the LCs, which are diagonally positioned in the *Dnali1*^*+/+*^ spermatozoa. **C** The co-staining and the co-localization analysis of AKAP3 and βTUBULIN (β-TUB). The red lines indicate the area where the fluorescence intensity are measured. **D** Quantitative co-localization analysis of AKAP3 and β-TUB. **E** IF staining against AKAP4. **F**–**I** IF staining depicting the locations of each DNAH proteins in the *Dnali1*^*+/+*^ and *Dnali1*^*−/−*^ mice. The asterisks indicate the miss of DNAH1 and DNAH7, respectively.

Furthermore, evidence from TEM revealed typical FS abnormalities in a majority of spermatozoa, similar to the proband (Fig. 4A). Specifically, the almost symmetrical LC distribution was altered to form an asymmetric distribution in the principal piece of most *Dnali1*^−/−^ mice flagella (Fig. 4B). Then, we detected the localization of two important structural proteins of FS, AKAP3, and AKAP4, in the sperm of *Dnali1*^−/−^ mice. AKAP3 protein signal showed obvious asymmetry in the sperm flagellum of *Dnali1*^−/−^ mice (Fig. 4C and D). And similar to the patient’s sperm, AKAP4 expression was significantly reduced (Fig. 4E). To reveal the potentially affected IDA-DHC components in *Dnali1*^−/−^ mice, flagellar immunostaining with anti-DNAH1\7\10\12 were performed. Consistent with the experimental observations in the proband, DNAH1, and DNAH7 signals were absent from the flagella lacking DNALI1, whereas, the DNAH10 signal was still obvious (Fig. 4F–I). Overall, the sperm phenotypes of the *Dnali1*^−/−^ mice were fully consistent with those of the *DNALI1*^*663_666del*^ patient, thereby allowing us to declare that DNALI1 deficiency indeed caused male infertility with AZS.

### DNALI1 protein localizes in the cytoplasm of testicular round spermatids and regulates the assembly of flagellar components

**Fig. 5:**
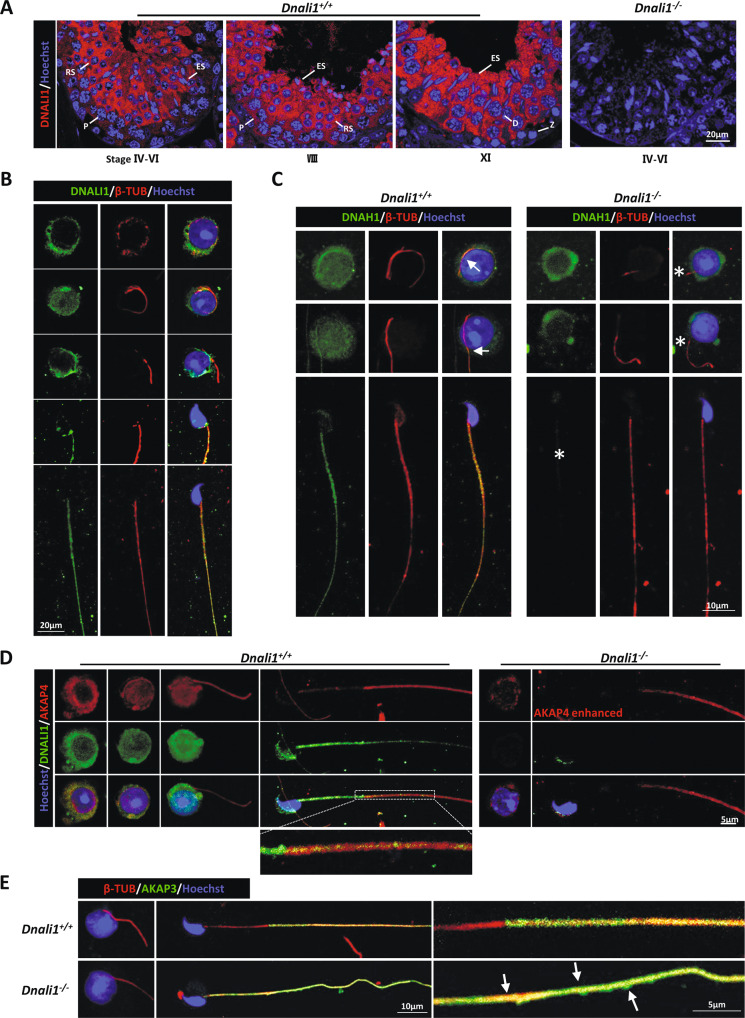
DNALI1 localizes in the cytoplasm of spermatids and regulates the assembly of structural proteins in the flagellum. **A** DNALI1 staining at distinct murine developmental phases of the male germ cells, as evidenced by IF. Z zygotene, P Pachytene, D Diplotene, RS round spermatid, ES elongated spermatids. **B** IF analysis of round and elongated spermatids from the *Dnali1*^*+/+*^ mice. **C** The expression and location of DNAH1 during spermiogenesis. The DNAH1 protein is expressed, but cannot be assembled into the flagella. Arrows indicate the localization of DNAH1 in the flagella, and the asterisks indicate the absence. **D** The AKAP4 staining during spermiogenesis. The AKAP4 expression and assembly efficiency are significantly reduced. **E** IF analysis of AKAP3 during spermiogenesis. Arrows mark the abnormal distribution of AKAP3.

To elucidate the significance of DNALI1 in flagellar assembly, we assessed DNALI1 expression during spermatogenesis. Consistent with a previous study [19], DNALI1 was detected in the cytoplasm during spermiogenesis. Furthermore, we detected strong signals in spermatocytes from the mid-pachytene to the metaphase II (Fig. 5A). In addition, IF staining of testicular single-cell suspension was performed. DNALI1 was expressed in the cytoplasm of round spermatids prior to flagellar formation. Notably, there were no DNALI1 signals on the flagella when it initially protruded from the cytoplasm. DNALI1 was reassembled after this process (Fig. 5B). These results suggested that DNALI1 may possess other functions, in combination with being a component of IDA.

To further explore the process of sperm flagellar assembly in KO mice, we detected the expression dynamics of DNAH1, AKAP3, and AKAP4 proteins. Strong DNAH1 signals were detected in the cytoplasm of *Dnali1*^*−/−*^ round spermatids, however, this signal did not extend to the flagella (Fig. 5C). This may be because DNALI1 is a LIC protein linked to DNAH1. The loss of DNALI1 did not affect DNAH1 expression, however, it hindered its anchoring to the flagella. In the meantime, levels of AKAP4, an FS protein, were significantly diminished in the cytoplasm and flagella of *Dnali1*^*−/−*^ spermatids, although it was still expressed (Fig. 5D). Similarly, the abnormal localization of AKAP3 appeared during flagellar elongation (Fig. 5E).

**Fig. 6:**
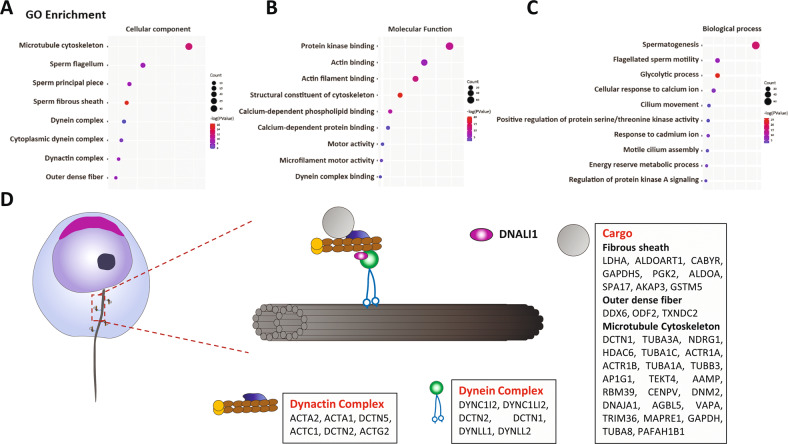
DNALI1 may serve as a portion of the cytoplasmic dynein complex that regulates the transport and assembly of flagellar components. **A**–**C** GO analysis of the DNALI1 binding proteins in mice testes. **D** A schematic of the cellular distributions of the DNALI1 binding proteins. The DNALI1 binding proteins are enriched in the Dynactin and Dynein complexes. A large number of flagellum component proteins are identified and may serve as cargos transmitted by the dynein complex.

To further explore the mechanism of the abnormal assembly of AKAP3 and AKAP4 in *Dnali1*^*−/−*^ flagellum, we identified the DNALI1-interacting protein in the testes using MS (Table S2). Gene ontology (GO) analysis of the associated proteins using DAVID tools functional annotation clustering identified enrichment of cellular components of the microtubule cytoskeleton, sperm flagellum, sperm fibrous sheath, and dynein complex (Fig. 6A). In terms of molecular functions and biological processes, DNALI1-interacting proteins were enriched in protein kinases, glucose metabolism, and ion channels, all of which are related to FS activity [7, 27]. In addition, motor activity and dynein complex binding were regulated by cytoplasmic transport-related proteins (Fig. 6B and C). Combined with the abnormal localization of AKAP3 and AKAP4, we hypothesized that DNALI1 may be involved in the regulation of cytoplasmic dynein, which, in turn, played an indispensable role in flagella, especially FS assembly (Fig. 6D).

### DNALI1 is a brain ciliary component, and loss of DNALI1 causes hydrocephalus in mice

**Fig. 7:**
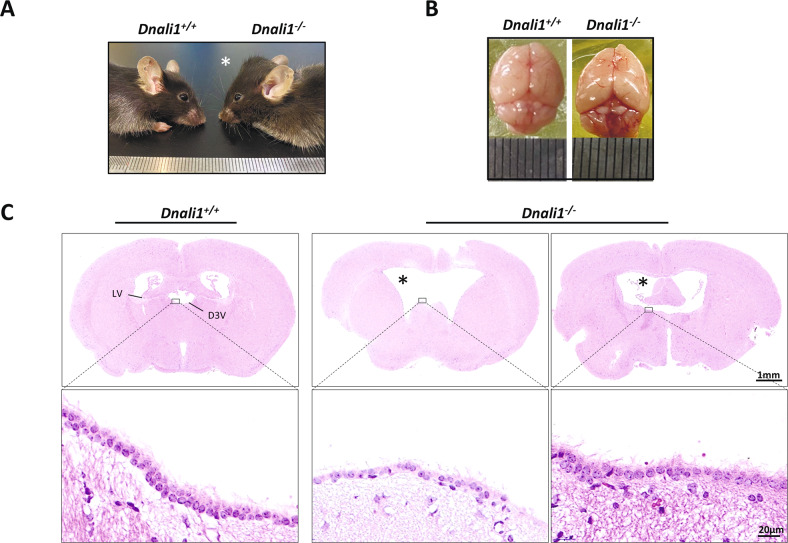
*Dnali1*^*−/−*^ mice have distinct hydrocephalus. **A** Representative lateral images of 8-week-old *Dnali1*^*−/−*^ mice with a domed cranial vault, compared to the *Dnali1*^*+/+*^ mice. **B** The top view of the whole skulls from the *Dnali1*^*+/+*^ and *Dnali1*^*−/−*^ mice. **C** The coronal mice brain sections stained with H&E. High magnification images reveal that cilia are present on intact *Dnali1*^*−/−*^ ependyma. D3V dorsal 3rd ventricle, LV lateral ventricle. The asterisk indicates the enlarged ventricle.

Being a critical member of the sperm flagella and cilia, IDAs loss can induce additional ciliopathies, on top of infertility, due to its ciliary abnormalities. We, therefore, examined whether other ciliopathy-associated phenotypes also exist in the *Dnali1*^*−/−*^ mice. Nearly half of the *Dnali1*^*−/−*^ mice, regardless of sex, developed obviously enlarged heads and hydrocephaly with complete penetrance (Fig. 7A and B). Accordingly, H&E staining verified the dilated cerebral ventricles in the affected *Dnali1*^*−/−*^ mice (Fig. 7C). IF staining revealed a strong DNALI1 signal in the ventricular cilia (Fig. S2C); however, we observed no DNALI1 expression in the respiratory cilia (Fig. S2A and B), suggesting a distinct DNALI1 expression in different cilia-enriched tissues.

### *DNALI1*^*663_666del*^ patient had a good prognosis following ICSI

ICIS was adopted to overcome primary infertility faced by the presented couple. Prior to ICSI, we sequenced *DNALI1* to eliminate the possibility of the partner carrying any deleterious mutations in the same gene. The couple then underwent one ICSI cycle and experienced a singleton pregnancy following the implantation of one freeze-thawed embryo. The oocyte fertilization and embryo development rates (eight cells and blastocyst) were 100.0%, 50.0%, and 50.0%, respectively (Table 1). Eventually, the woman delivered a healthy baby boy with a full-term pregnancy. This evidence indicated that ICSI was effectively employed as a reproductive approach for our *DNALI1*^*663_666del*^ patient.

## Discussion

Dynein motors-based bending and twisting of the flagellar axoneme provide the propulsive force for sperm movement. In this dynamic machinery, the axonemal DMTs act as a scaffold, upon which the fixed outward ODAs and inward IDAs bridge the inter-doublet gap, and drive the adjacent parallel DMTs to slide past one another. Of note, the IDAs underlie the primary motors that generate the beating waveform, while the ODAs provide an additional force that coordinates this event [38], highlighting the irreplaceability of IDAs in regulating flagellar motility. The IDA apparatus is highly conserved in evolution and comprises three complexes that are longitudinally arranged within a 96-nm axonemal repeat along the length of DMTs [39]. Each IDA complex contains several subunits with distinctive molecular weights and cellular functions. The distal HDC subunits possess ATPase activity, which converts ATP hydrolysis-based chemical energy to mechanical force that drives the microtubular movement. In comparison, the proximal light subunits, including IC, LC, and LIC, construct the base of the IDA complex and are expected to be vital in regulating dynein activity [40]. This conclusion is strongly supported by the conclusions of the human ciliopathy-based genetic investigation. Defects in *DNAI1*, *DNAI2*, and *DNAI3*, which encode the IC subunit of axonemal dyneins, impair the integrity of dynein motors, thus, resulting in dyskinetic respiratory cilia or sperm flagella [41–43].

The mammalian *DNALI1* is presumed to encode a LIC subunit orthologous to p28, which is an integral part of the IDAs in *Chlamydomonas*. As anticipated, the murine DNALI1 is localized within the entire sperm flagellum and interacts with the HDC subunit DNAH1 in the testis [19]. Furthermore, human *DNALI1* is also mainly expressed in the testis [18]. Despite this evidence indicating the potential significance of DNALI1 in modulating flagellogenesis, they sidestep the question of whether DNALI1 deficiency causes sperm dyskinesia and male infertility and, if so, what underlying mechanisms are behind it. Herein, we detected a rare homozygous frameshift mutation in *DNALI1* [c.663_666del (p.Glu221fs)] in a Chinese infertile AZS patient with consanguineous parents. The complete loss of DNALI1 in the mutant spermatozoa demonstrated the pathogenicity of this putative LOF mutation. Additionally, our *Dnali1*-KO mice also displayed a typical ASZ phenotype. These genetic and phenotypic analyses in both human and murine species provide corroborating evidence to conclude that *DNALI1* is a newly recognized causative gene for AZS in both aforementioned species.

Although there was no evidence linking the DNALI1 deficiency with MMAF, TEM analyses revealed unique ultrastructural defects in FS in the *DNALI1*^*663_666del*^ patient. Specifically, the irregularly distributed LC accounted for over 79% of the flagellar cross-sections from the principal piece, which was in line with the typical LC alterations observed in the *Dnali1*^*−/−*^ mice. The dramatically decreased AKAP4 and anomalously distributed AKAP3 in the DANLI1 deficiency flagella, combined with our proteomic findings in mice that multiple FS-associated proteins are potentially connected with DNALI1 during spermatogenesis, we established a potential function of DNALI1 in modulating the molecular assembly and biological activity of FS. Based on the subcellular structural evidence from *Chlamydomonas* and Sea urchin sperm flagella, DNALI1 might combine with specific HDC subunits, such as DNAH1, DNAH7, and DNAH12, to form the corresponding IDAs in mammals [37]. Accordingly, we demonstrated that DNAH1 and DNAH7 were absent from the flagella lacking DNALI1 in both species. Moreover, based on the proteomic analyses of human spermatozoa, both DNAH1 and DNAH12 potentially interacted with DNALI1, which further reinforced this hypothesis. On the contrary, DNALI1 persisted in the flagella with a deficiency in either DNAH1 or DNAH7. These findings unequivocally suggested that DNALI1 can function as a fundamental component in determining the flagellar localization of the relevant HDC subunits during the assembly of several specific IDAs in mammals. Therefore, it is tempting to speculate that certain axonemal IDAs were not impaired by the absence of DNALI1, which reasonably explained why there were no apparent alterations in the ultrastructure of IDAs in our patient and KO mice. In fact, the visible DNAH10 and DRCI IF signals in the *DNALI1*^*663_666del*^ flagella strongly supported this viewpoint. Higher resolution and in-depth structures of IDAs are warranted to test these hypotheses. Collectively, the subcellular abnormalities in the flagellar FS and IDAs, caused by the DANLI1 deficiency, might underlie the etiology of AZS in humans and mice.

Besides the beating of axonemes, the dynein family also participates in the intracellular transport of cargoes, termed cytoplasmic dynein (dynein-1). Dynein-1 forms a DDX complex with dynactin to transport diverse cargoes, such as lysosomes, peroxisomes, and phagosomes, towards the negative end of the cytoplasmic microtubules in a broad range of biological processes [44]. Our proteomic analyses revealed the potential association between DNALI1 and both IC/LIC subunits of the dynein-1 [45], as well as multiple subunits of the dynactin complex. This evidence, combined with our IF findings on the initial expression of DNALI1 in the cytoplasm of postmeiotic germ cells before its distribution within the mature spermatozoa flagella, raised an interesting potential that DNALI1 might serve as an essential coordinator of the DDX complex regulating the cytoplasmic transport of flagellogenesis-based cargoes during sperm morphogenesis. This amazing assumption is supported to some extent by our experimental data. For instance, AKAP4 was markedly reduced in both the spermatid cytoplasm and sperm flagella in the *Dnali1*^*−/−*^ mice, suggesting that the cytoplasmic transport efficiency of AKAP4 was seriously impaired in the absence of DNALI1, which eventually disrupted the subsequent flagellar assembly of AKAP4. Furthermore, the flagellar distribution of AKAP3 was apparently asymmetrical in the *Dnali1*^*−/−*^ mice, which also indicated that the accuracy of AKAP3 assembly was disrupted without DNALI1. These results collectively revealed a potential non-classical molecular function of DNALI1 in modulating spermatogenesis, especially the FS assembly. However, this preliminary conclusion requires further verification in future investigations.

Axonemal defects always cause ciliopathy in mammals [46]. In addition to the testicular tissue, we also identified DNALI1 in the ventricular cilia, rather than in the respiratory cilia. The distinct DNALI1 expressions in different cilia-enriched tissues implied possible clinical features and pathophysiology of DNALI1 deficiency. As we previously reported in the *Drc1*^*−/−*^ mice [26], we identified hydrocephaly in nearly half of the *Dnali1*^*−/−*^ mice, which may have resulted from ventricular ciliary dysfunction. The phenotypic differences, other than sterility, between the proband and KO mice may have been due to their different genetic backgrounds. Till date, voluminous empirical evidence revealed that AZS-affected individuals experience a desirable outcome after ICSI, particularly for patients harboring defects in the axonemal genes [47]. As expected, the *DNALI1*-mutant patient successfully obtained his biological offspring using ICSI, which is informative for the personalized recommendation of reproductive management.

In summary, we demonstrated, for the first time, that DNALI1 is critical for the normal subcellular architecture and motor function of sperm flagellum in humans and mice. Our thorough investigation further revealed the significance of DNALI1 in regulating IDAs assembly, and in coordinating the cytoplasmic transport of flagellar components during spermatogenesis. This study expanded our knowledge of the genetic etiology and clinical guidance for human AZS.

## Supplementary information

Supplementary Figure and Table legends

The mutant site of DNALI1 is highly conserved in multiple species.

The expression pattern of DNALI1 in lung, trachea and ventricular cilia of mice.

Spermatozoa collected from Dnali1+/+ mice.

Spermatozoa collected from Dnali1−/− mice.

DNALI1 interacting proteins in the human sperm

DNALI1 interacting proteins in the mice testes

uncropped Western blot images

## Data Availability

The original data produced in this study are available from the corresponding author upon request. Mass spectrometry proteomics data have been deposited to the ProteomeXchange Consortium via the PRIDE partner repository with the dataset identifier PXD039301 and PXD039300.
