# Supplementary material for: DNALI1 deficiency causes male infertility with severe asthenozoospermia in humans and mice by disrupting the assembly of the flagellar inner dynein arms and fibrous sheath
Source: Cell Death Dis. 2023 Feb 15;14(2):127. doi: 10.1038/s41419-023-05653-y (PMC9932082; doi:10.1038/s41419-023-05653-y)
Supplement: Supplementary file 1 — Supplementary Figure and Table legends [file 41419_2023_5653_MOESM1_ESM.docx]

**Supplementary Figure and Table legends**

**Supplementary Figure S1. The mutant site of DNALI1 is highly conserved in multiple species.**

**(A)** An illustration of the domains of DNALI1 protein product, based on the NCBI browser. The positions of the novel DNALI1 mutation introduced in this study are marked with black dotted lines. **(B)** Sequence alignment of DNALI1 proteins from primates, rodents, Cypriniformes, and Artiodactyla. The red box marks the amino acid sites where frameshift occurs.

**Supplementary Figure S2. The expression pattern of DNALI1 in lung, trachea and ventricular cilia of mice.**

**(A)** Western blot analysis of protein extracts from the lung and testes. β-TUBULIN (β-TUB) served as the loading control. No specific bands was detected in the lung. **(B)** - **(C)** Tracheal **(B)** and ventricular **(C)** cilia were stained with the indicated antibodies. IF staining revealed a strong DNALI1 signal in the ventricular cilia **(C)**; no specific DNALI1 signal was detected in the tracheal cilia **(B)**.

**Supplementary** **Table S1.** DNALI1 interacting proteins in the human sperm.

**Supplementary Table S2.** DNALI1 interacting proteins in the mice testes.

**Supplementary** **Movie S1.** Spermatozoa collected from *Dnali1^+/+^* mice.

**Supplementary** **Movie S2.** Spermatozoa collected from *Dnali1^-/-^* mice.
