# Supplementary figures and images for: DNALI1 deficiency causes male infertility with severe asthenozoospermia in humans and mice by disrupting the assembly of the flagellar inner dynein arms and fibrous sheath
Source: Cell Death Dis. 2023 Feb 15;14(2):127. doi: 10.1038/s41419-023-05653-y (PMC9932082; doi:10.1038/s41419-023-05653-y)

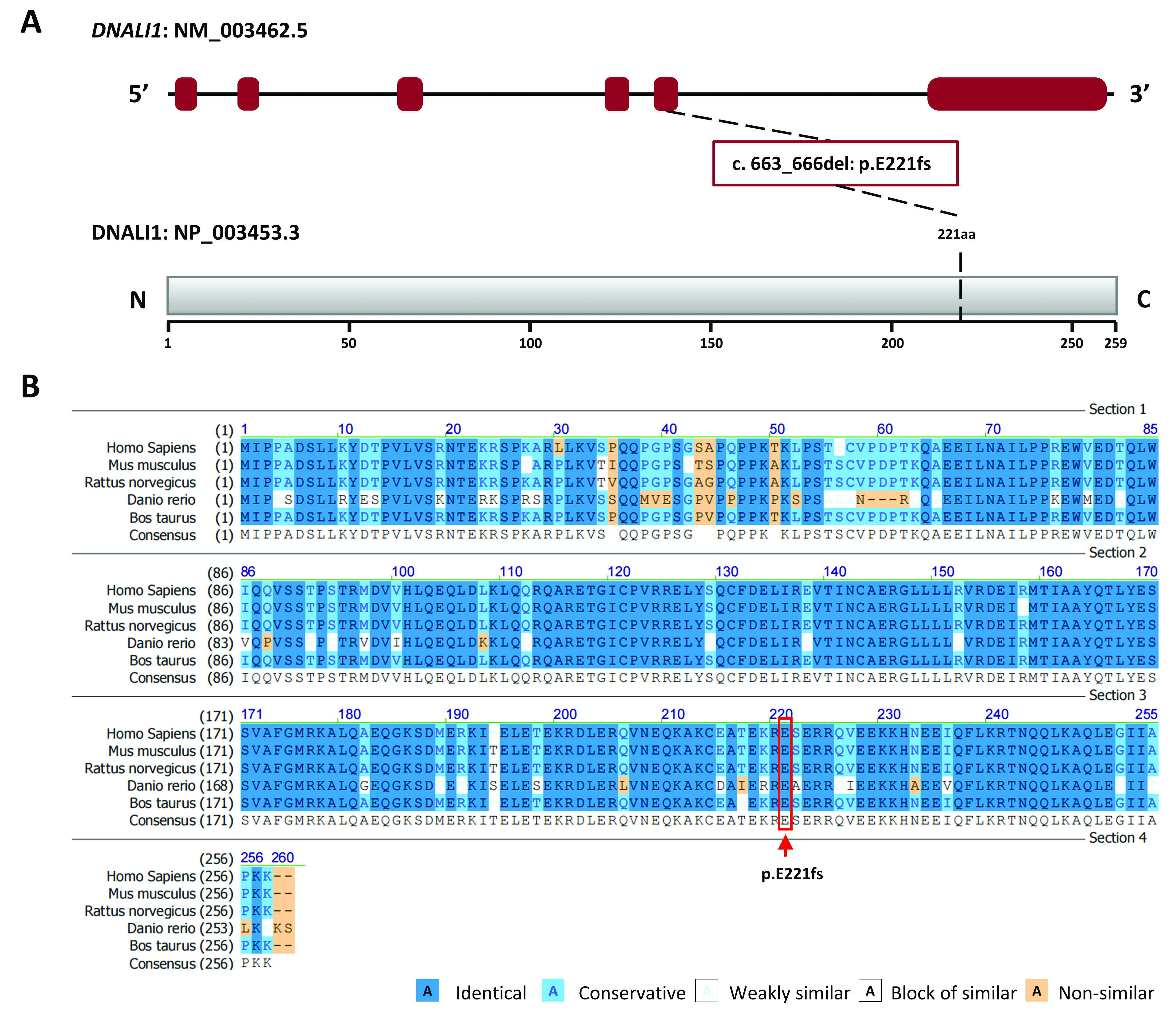

Supplement: Supplementary file 2 — The mutant site of DNALI1 is highly conserved in multiple species. [file 41419_2023_5653_MOESM2_ESM.tif]

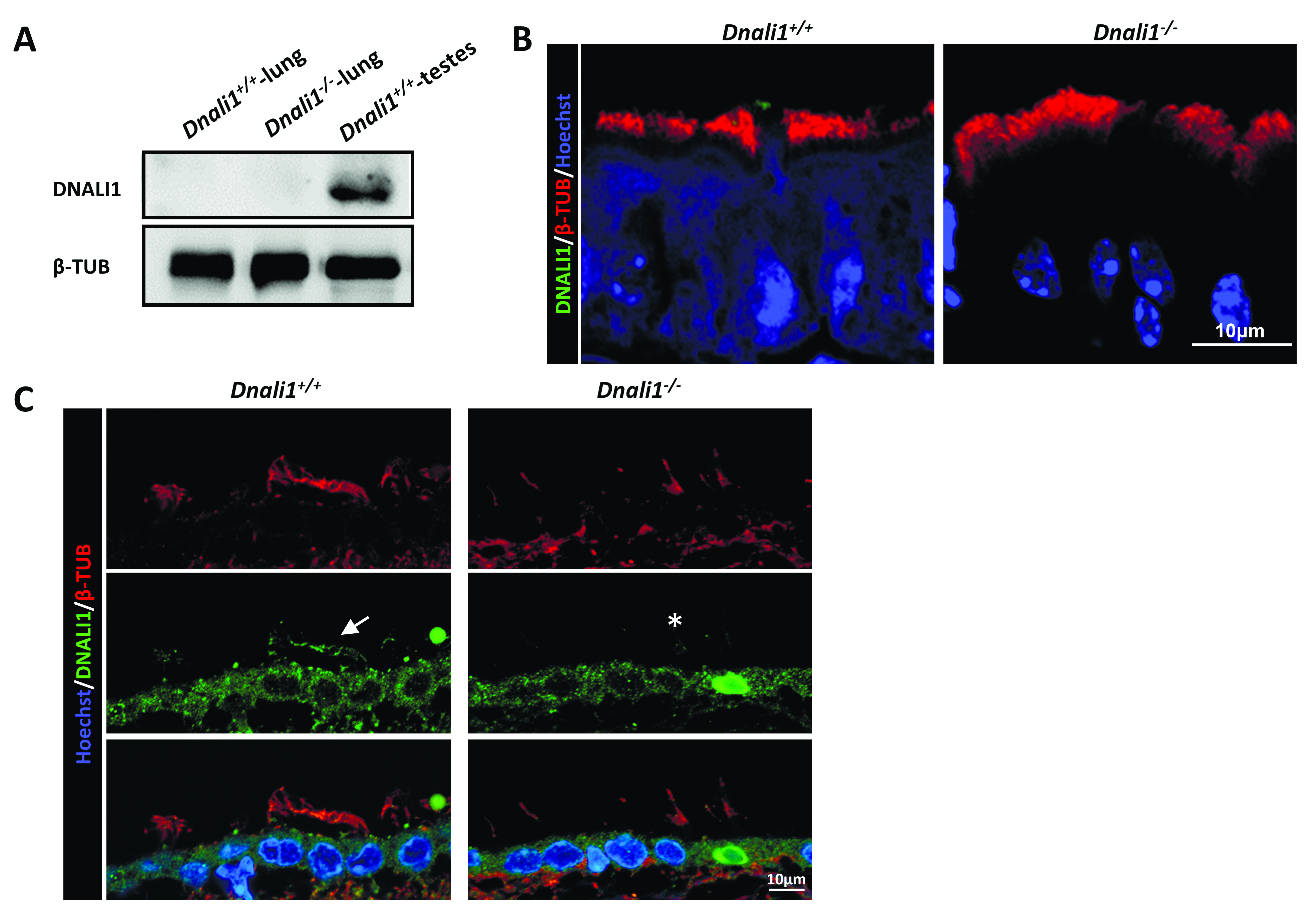

Supplement: Supplementary file 3 — The expression pattern of DNALI1 in lung, trachea and ventricular cilia of mice. [file 41419_2023_5653_MOESM3_ESM.tif]

Related to Figure 1C

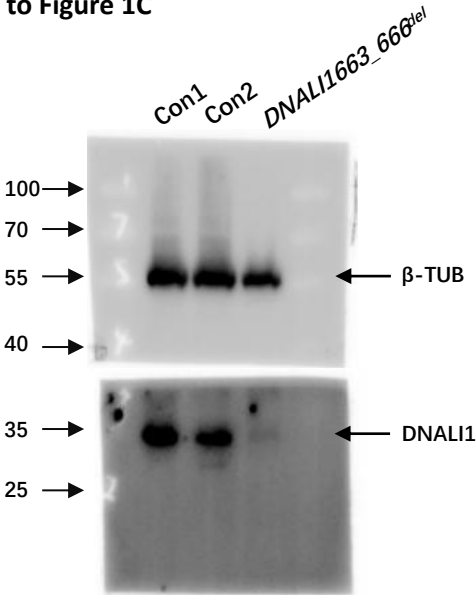

Related to Figure 2C

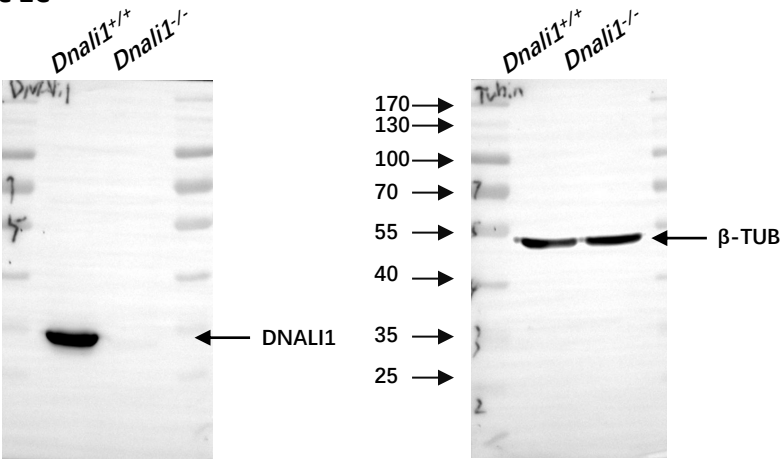

Supplement: Supplementary file 8 — uncropped Western blot images [file 41419_2023_5653_MOESM8_ESM.pdf]
